# Strontium isoscapes for provenance, mobility and migration: the way forward

**DOI:** 10.1098/rsos.250283

**Published:** 2025-06-18

**Authors:** Maximilian J. Spies, Amanda Alblas, Stanley H. Ambrose, Sarah Barakat, Ramiro Barberena, Clément Bataille, Gabriel J. Bowen, Kate Britton, Hayley Cawthra, Roger Diamond, Anthony Dosseto, Jane A. Evans, Erich Fisher, Kerryn Gray, Phoebe Heddell-Stevens, Emily Holt, Hannah F. James, Anneke Janzen, Mael Le Corré, Petrus le Roux, Julia Lee-Thorp, Alexander Mackay, Patricia J. McNeill, Janet Montgomery, Bedone Mugabe, Vicky M. Oelze, Michèle Pfab, Michael P. Richards, Celeste T. Samec, Francisca Santana-Sagredo, Alejandro Serna, Chris Stantis, Christophe Snoeck, Brian Stewart, Cameron Stuurman, Damon Tarrant, Adam G. West, Christine Winter-Schuh, Judith Sealy

**Affiliations:** ^1^ Department of Archaeology, University of Cape Town, Cape Town, South Africa; ^2^ Division of Clinical Anatomy, Faculty of Medicine and Health Sciences, Stellenbosch University, Cape Town, South Africa; ^3^ Department of Anthropology, University of Illinois, Urbana, IL, USA; ^4^ Department of Archaeology, University of Aberdeen, Aberdeen, UK; ^5^ Centro de Investigación, Innovación y Creación (CIIC), Universidad Católica de Temuco Facultad de Ciencias Sociales y Humanidades, Temuco, Araucania, Chile; ^6^ Instituto Interdisciplinario de Ciencias Básicas (ICB), Consejo Nacional de Investigaciones Científicas y Técnicas (CONICET), Facultad de Ciencias Exactas y Naturales, Laboratorio de Paleoecología Humana, Universidad Nacional de Cuyo, Mendoza, Argentina; ^7^ Department of Earth and Environmental Sciences, University of Ottawa, Ottawa, Ontario, Canada; ^8^ Department of Forestry and Natural Resources, Purdue University, West Lafayette, IN, USA; ^9^ Department of Geology and Geophysics, University of Utah, Salt Lake City, UT, USA; ^10^ Minerals and Energy Unit, Council for Geoscience, Cape Town, South Africa; ^11^ African Centre for Coastal Palaeoscience, Nelson Mandela University, Gqeberha, South Africa; ^12^ Biogeochemistry Research Infrastructure Platform (BIOGRIP), University of Cape Town, Cape Town, South Africa; ^13^ Wollongong Isotope Geochronology Laboratory, School of Earth and Environmental Sciences, University of Wollongong, Wollongong, New South Wales, Australia; ^14^ British Geological Survey, Nottingham, UK; ^15^ Centro Interdisciplinar de Arqueologia e Evolução do Comportamento Humano, Universidade do Algarve, Faro, Portugal; ^16^ Department of Geological Sciences, University of Cape Town, Cape Town, South Africa; ^17^ Department of Archaeology, Max Planck Institute of Geoanthropology, Jena, Germany; ^18^ School of History, Archaeology, and Religion, Cardiff University, Cardiff, UK; ^19^ Department of Archaeology, Environmental Changes and Geo-Chemistry, Vrije Universiteit Brussel, Brussels, Belgium; ^20^ Department of Anthropology, University of Tennessee Knoxville, Knoxville, TN, USA; ^21^ BioArchéologie, Interactions Sociétés environnements (BioArch—UMR 7209), Muséum National d’Histoire Naturelle, Paris, France; ^22^ School of Archaeology, University of Oxford, Oxford, England, UK; ^23^ Environmental Futures Research Institute, School of Science, University of Wollongong, Wollongong, Australia; ^24^ Paleoanthropology, University of California Davis, Davis, CA, USA; ^25^ Department of Archaeology, Durham University, Durham, UK; ^26^ Department of Anthropology, University of California Santa Cruz, Santa Cruz, CA, USA; ^27^ South African National Biodiversity Institute (SANBI), Pretoria, South Africa; ^28^ Department of Archaeology, Simon Fraser University, Burnaby, British Columbia, Canada; ^29^ Instituto de Geocronología y Geología Isotópica (CONICET-UBA), Universidad de Buenos Aires, Buenos Aires, Argentina; ^30^ Escuela de Antropología, Pontificia Universidad Católica de Chile, Santiago, Chile; ^31^ Department of Archaeology, University of York, York, UK; ^32^ División Arqueología, Facultad de Ciencias Naturales y Museo, Universidad Nacional de la Plata, La Plata, Buenos Aires, Argentina; ^33^ School of Anthropology, Political Science, and Sociology, Southern Illinois University Carbondale, Carbondale, IL, USA; ^34^ Department of Anthropology, University of Michigan, Ann Arbor, MI, USA; ^35^ Department of Biological Sciences, University of Cape Town, Cape Town, South Africa; ^36^ Institute for Prehistory and Early History, Christian-Albrechts-University zu Kiel, Kiel, Germany

**Keywords:** geolocation, isotope mapping, geostatistics, ecology, geochemistry, bioavailable ^87^Sr/^86^Sr

## Abstract

Strontium isotopes (^87^Sr/^86^Sr) are increasingly used as a provenance tool in multiple disciplines. Application to biological materials requires knowledge of the variation in bioavailable ^87^Sr/^86^Sr across the landscape, potentially in the form of an isoscape (a quantitative model of spatial isotopic variability). This paper summarizes and provides advice on our current understanding of the main concerns in creating and interpreting isoscapes of bioavailable ^87^Sr/^86^Sr. Isoscape creation approaches include domain mapping, geostatistical contour mapping and machine learning, the last becoming more readily achievable with the availability of software packages. It is critically important to develop isoscapes at a resolution appropriate for addressing the research questions. Choice of sample materials depends on the research questions and availability: plants or fauna with small ranges are favoured, with some analytes (snails, soil leachates) posing challenges. Interpreting ^87^Sr/^86^Sr in biological tissues requires considering Sr metabolism and the timing of tissue formation, thus far underappreciated. The numerous sources of error involved in developing and applying isoscapes must be recognized to avoid over-interpreting data and spurious provenance precision. We hope this paper will help researchers investigating provenance, mobility, landscape use and migration to develop the most appropriate isoscapes for their purposes, and possible future use by others.

## 1. Introduction

Strontium isotopes (^87^Sr/^86^Sr) are increasingly widely used as a provenance tool across many fields, including archaeology, ecology, biology, forensics and the food industry (e.g. [1–16]). In recent years, there has been substantial development in the field, especially improvements in the creation of quantitative spatial models of isotopic variation, or ‘isoscapes’, and the use of probabilistic models for geographic allocation. Yet, some important challenges remain, many of which were identified in reviews by Holt *et al.* [17] and Bataille *et al.* [18]. In January 2024, the authors of this paper held a five-day meeting in Cape Town, South Africa, to workshop these issues, discuss best practices in the construction and interpretation of isoscapes, and flag aspects requiring further research. This article summarizes the outcomes of those discussions. We hope it will be useful to the growing community of researchers using strontium isotope provenancing worldwide.

The application of ^87^Sr/^86^Sr as a provenance tool is based on the ratios of strontium and rubidium isotopes in the local environment, which in most cases derive from the underlying local geology. ^87^Sr is the stable radiogenic daughter isotope of ^87^Rb (*t*_½_ = 48.8 × 10^9^ years), while ^86^Sr is stable and largely invariant over time. Older rocks and those rich in ^87^Rb show relatively high ^87^Sr/^86^Sr, while younger (e.g. recent volcanic) substrates have lower values. Although bioavailable ^87^Sr/^86^Sr (i.e. that taken up by plants and animals) can differ from that of bedrock, these ratios are persistent in soils derived from parent substrates (albeit with other inputs, and outputs—see below), in the tissues of the plants growing in those soils, the herbivores feeding on those plants, and the predators preying on them. The single mass unit difference between ^87^Sr and ^86^Sr is small in relation to total mass, so there is little mass-dependent fractionation of these isotopes along the food chain, though see [19,20]. The small amount that does occur is corrected for by adjusting all measured ^87^Sr/^86^Sr to a constant value of 0.1194 for ^86^Sr/^88^Sr [21]. The ^87^Sr/^86^Sr of consumers should therefore track the mixing of ingested food (and in some cases water) with distinct ^87^Sr/^86^Sr, taking into account differences in strontium concentrations of each Sr source, and possible differential digestion and absorption of each source within the digestive tract of species with differing gut pH [22,23]. ^87^Sr/^86^Sr in the environment may differ from bedrock ratios if the geology is heterogeneous and contains minerals with differing ^87^Sr/^86^Sr and varying susceptibilities to weathering, such as granite and sandstone [24–26]. In addition, soils (and therefore plants and animals) can incorporate Sr from sources other than bedrock, such as atmospheric, fluvial and anthropogenic sources. Atmospheric inputs include precipitation and wind-transported dust, marine aerosols and volcanic ash (e.g [26–29]). The importance of these inputs depends on the magnitude of the fluxes, and the levels of endogenous bedrock-derived strontium in the soils. Coastal regions may have ^87^Sr/^86^Sr dominated by Sr from seawater-derived aerosols and precipitation or geologically recent carbonate-rich sands draped over underlying geology [4,26,30–32]. This influence can extend hundreds of kilometres inland [33]. Rivers flowing through different geological formations may contain dissolved Sr and/or contribute sediments with ^87^Sr/^86^Sr that differs from local bedrock [24,34–36]. Anthropogenic activities, such as quarrying, mining, soil transport and the addition of agricultural lime, can substantially alter soil ^87^Sr/^86^Sr [37–39] and introduce greater variation through the soil profile [40]. These and similar processes may cause bioavailable ^87^Sr/^86^Sr to be decoupled from that of local bedrock [41,42].

Multiple possible Sr inputs at different scales make isoscapes of ^87^Sr/^86^Sr more challenging to develop and use than (for example) isoscapes of ^18^O/^16^O (δ^18^0) and ^2^H/^1^H (δ^2^H) in precipitation, in which variation driven by large-scale atmospheric processes leads to more consistent patterning [43]. For these reasons, ^87^Sr/^86^Sr provenancing in many regions cannot be undertaken with reference to geological maps alone and requires the development of isoscapes, or predictive models of geospatial variation in ^87^Sr/^86^Sr [44]. In other words, isoscapes are not simply maps of empirical data, they are sets of expectations (mathematical predictions) derived from both first principles and a limited amount of empirical data. They tend to preferentially emphasize overall patterning over the degree of variation at individual localities. Isoscapes enable us to visualize and model ^87^Sr/^86^Sr across a landscape, incorporating multiple different Sr sources. Strontium isoscapes are usually generated from baseline reference material (e.g. plants) and then used to track the mobility of animals or humans by matching ^87^Sr/^86^Sr from their tissues to the isoscape. This approach works well due to negligible trophic fractionation of ^87^Sr/^86^Sr, and the stability of these ratios over time scales relevant to ecology, forensics, archaeology and related disciplines. There are, however, several challenges, which we examine below.

First, it is important to note that ^87^Sr/^86^Sr will not address all questions about mobility and provenance. Modelling the distribution of ^87^Sr/^86^Sr requires considerable laboratory and computing effort and is not to be undertaken lightly. Researchers should first carefully consider whether there is a reasonable expectation that the spatial patterning in ^87^Sr/^86^Sr is likely to be capable of addressing the particular research question, given the nature of the landscape and its geological formations.

Below, we explore key questions that formed the main topics of discussion in our 2024 workshop. Many of us have found these difficult to resolve in our own work. Holt *et al.* [17] provide an excellent overview of the field. Based on their review and other recent papers, we selected the following topics: the relative merits of different approaches to isoscape development, sample collection strategies, choice of sample type and the requisite preparation and analysis, complications with using isoscapes to assign provenance, including the influence of consumer biology and metabolism, whether it is necessary to tailor isoscapes for specific applications, uncertainties and limitations and future directions for the field. These topics span both fundamental principles and laboratory practice. The choices researchers make may differ depending on the field of study and the specific research questions, but we hope that the application of these principles will guide practitioners towards better outcomes.

## 2. Approaches to isoscape development: are machine-learning isoscapes better than other types?

The framing of this question, and some of the section headings below, may appear to be leading the reader towards particular conclusions. These are, however, informed by careful reading of the current literature, which guided our discussions. We believe that this more targeted approach helps discriminate between various possible options and leads to a clearer understanding of current best practice.

There are currently three main approaches to developing isoscapes of bioavailable ^87^Sr/^86^Sr, each with advantages and disadvantages. The first, domain mapping, is based on clustering empirically measured ^87^Sr/^86^Sr (normally bounded or grouped by lithology), while the second and third, geostatistical and machine learning approaches, respectively, use empirical ^87^Sr/^86^Sr measurements alongside other environmental datasets to model and predict ^87^Sr/^86^Sr variation. These approaches have been described [17,45] and compared elsewhere [45–47], but for completeness, we outline the basics of each below.

Domain mapping is a clustering technique that groups empirical ^87^Sr/^86^Sr data derived from different user-defined domains, generally geological units [17]. Each unit is then assigned a median and range. This is an easy, conservative and fast approach to visualizing patterns of bioavailable ^87^Sr/^86^Sr, but it depends heavily on good sample coverage and the assignment of the domains [17]. If these are geological units, the assumption is that geology is the dominant driver of ^87^Sr/^86^Sr, and the quality of the isoscape will depend on the quality and resolution of geological maps used to delineate domains, as well as careful field observations determining the context of each sampled site. If there are other influences on ^87^Sr/^86^Sr, these are captured in the form of large uncertainties per domain. This method is, therefore, most useful in regions where lithology plays the dominant role and is well-mapped, and other Sr inputs are limited. Domains can be defined based not only on lithological units but, for example, on proximity to the coast, where ^87^Sr/^86^Sr is likely to be similar across different geological substrates because of marine-derived Sr inputs [48,49]. Similarly, specific domains can be created to encompass (for example) old-growth forests, where the more soluble (e.g. carbonate-based) Sr components of the soil may be leached out and/or sequestered in the vegetation, so the bioavailable ^87^Sr/^86^Sr signal becomes dominated by the less-soluble soil component [50]. The strontium isoscape of Great Britain, available through the British Geological Survey, was created using domain mapping (biosphere isotope domains). The isoscape, the data on which it is based and various tools, including a useful query function, are all freely available at https://www.bgs.ac.uk/datasets/biosphere-isotope-domains-gb/.

The second approach uses various geostatistical methods, such as inverse distance weighting and kriging, to develop an output that can be visualized as a contour map. This enables continuous predictions of ^87^Sr/^86^Sr variation derived from measured known-origin samples [17]. Such maps are based on the principle of spatial autocorrelation, in which points closer together are considered to be more closely related (i.e. ratios will be more similar) than those further away. The simplest such method, inverse distance weighting (IDW), simply uses the distance between a locality with unknown ^87^Sr/^86^Sr and each point with a known value to weight their influences on the prediction. The scaling between distance and weight of influence in IDW is either assumed *a priori* or optimized by comparing predictions against testing data. Alternatively, kriging methods predict ^87^Sr/^86^Sr at a particular location using a variogram model to account for trends in the spatial autocorrelation between pairs of points in the dataset, and assign weights to the observations. In contrast to IDW, the variogram model (and thus weights used in kriging prediction) is fitted to the known-origin data. There are several types of kriging. Simple kriging assumes a known constant mean autocorrelation, while ordinary kriging assumes an unknown constant mean, which is locally estimated. Universal kriging (or kriging with external drift) incorporates second-order effects by estimating a trend based on an auxiliary predictor and uses this instead of the local mean used in ordinary kriging [47]. Empirical Bayesian kriging automates parameter selection and accounts for the error of the semivariogram. Co-kriging incorporates one or more secondary datasets spatially correlated with ^87^Sr/^86^Sr distributions (e.g. soil, plant and water ^87^Sr/^86^Sr datasets [46]) to try to increase prediction precision. However, this also introduces more variability due to the additional estimations of the autocorrelation for the secondary variable, as well as the cross-correlation between variables. These geostatistical approaches include uncertainty assessments that allow one to assess the probability of a correct prediction [45]. One major limitation of these methods is that they assume that patterns of variation in ^87^Sr/^86^Sr are continuous across the landscape. Although geostatistics-based isoscapes are relatively easy and quick to create and can be readily extrapolated to adjacent regions, they cannot accurately represent discrete, nonlinear patterns of bioavailable ^87^Sr/^86^Sr (e.g. across heterogeneous geology), even when co-kriging with a geological map.

The third approach, the use of machine learning models, particularly random forest regression, is the most recent development. In this method, single decision trees play a central role. Each decision tree uses predictor variables to split the empirical dataset of ^87^Sr/^86^Sr into groups of similar values. The tree does this by making a series of decisions based on the variability within each predictor variable [18,45,51]. The potential predictor variables can be anything that may affect ^87^Sr/^86^Sr, such as geology, distance from the coast, dust, volcanic ash deposition, precipitation and others. Although single decision trees can theoretically be used in isolation, they are prone to overfitting and instability, where even slight changes in the training data can result in significant changes to the model. Random forest algorithms address these issues by creating an ensemble of multiple decision trees. Each tree is trained in parallel using a random subset of the training data and predictor variables, a process known as ‘bagging’ [52]. The performance of the model is validated through *n*-fold cross-validation with the possibility for spatial partitioning of the testing set to avoid geographic biases. This means that *n* random subsets of the data are removed prior to model development and used to test the created model. The results are averaged to produce a final model that is less prone to overfitting, more stable and more robust. This can be done multiple times with different, random test sets; however, there is a trade-off as fewer data are then available for model development. This testing process need not be uniform or truly random: it is possible to target ‘problem’ areas with known low accuracy based on prior knowledge, areas with low sampling density or specific areas of interest based on the research question. Initially, random forest models make no assumptions about which predictor variables are the main drivers of ^87^Sr/^86^Sr variation, and all variables are incorporated into the model. However, one can use variable selection algorithms such as variable selection using random forests (VSURF) [53] to select the most suitable predictors for a given study area (i.e. the predictors with the strongest effect on the model’s predictive power). VSURF uses an iterative simulation process, constructing many random forest models but selectively removing specific variables to quantify their effect on the model performance. Through this process, the algorithm iteratively removes the least relevant predictor variables based on their lack of impact on the model performance, and finally refines the variable selection by removing any redundant predictor variables, see [53]. The relationship between predictors and ^87^Sr/^86^Sr can be conveniently visualized through partial dependence plots. Using the most relevant selected variables, the random forest algorithm is then tuned to build the most parsimonious and best-performing random forest model integrating selected geospatial predictors.

Random forest modelling is an effective method for mapping bioavailable ^87^Sr/^86^Sr, in many situations outperforming other modelling approaches [45]. Compared to domain mapping and geostatistical approaches, random forest has several advantages for predicting ^87^Sr/^86^Sr. It allows seamless integration of multidimensional data (e.g. points, categorical geological maps and continuous rasters of climate variables), and it copes well with outlying ^87^Sr/^86^Sr values. This approach accurately predicts the multiscale patterns of ^87^Sr/^86^Sr, resolving some of the limitations of contour mapping and domain mapping techniques. Although still reliant on empirical ^87^Sr/^86^Sr data, since random forest models incorporate additional correlated predictor variables, they can perform adequately in areas with low sample coverage [17]. However, as random forest models become more complex, they incorporate more predictors and require more empirical ^87^Sr/^86^Sr data, which can be difficult to obtain and can reach the limits of computing power on personal computers. At this point, they require cluster computing resources.

Additionally, the random forest framework does not account for spatial autocorrelation within the calibration dataset [54], limiting its ability to handle geographic sampling biases. Predictions and spatial uncertainty are not influenced by the location of the sampling sites, and regional bias might be introduced by sampling some areas more heavily than others. Recent studies using random forest models to map soil properties have proposed several approaches to overcome this issue. Sampling distribution can be accounted for by introducing geographic features as covariates in the model [55,56], and local variation can be integrated by combining multi-scale random forest models [57] or by weighting the prediction according to the distance from the nearest sampling sites [58]. There are also advantages in integrating multiple algorithms, including random forest, through ensemble machine learning to improve the accuracy and reduce the errors of the predictions [55,59]. While random forest models rely on a single algorithm, ensemble approaches use a set of methods that combine the predictions of multiple individual models, improving the performance and robustness of the final model [60]. The *landmap* package in R [61] provides methods for spatial prediction using ensemble machine learning and accounts for spatial auto-correlation by using oblique geographic coordinates as covariates [56]. Applied to bioavailable ^87^Sr/^86^Sr mapping in Eastern Canada, the ensemble approach provided similar predictions to the classic random forest model but with reduced spatial uncertainty, particularly in highly radiogenic areas with high uncertainty [54]. Although powerful, ensemble approaches that integrate local variation require a good spatial distribution of samples and more computing power than traditional random forest methods, which may limit their application to large-scale ^87^Sr/^86^Sr isoscapes.

## 3. What is the best sample collection methodology for creating isoscapes?

Sample collection strategies should be shaped by the chosen isoscape approach, the research questions to be answered, and the scale, resolution and budget of the project. Although systematic sampling (e.g. on a grid) may be desirable (e.g. [62]), this is not always practical or possible. Topography may limit access to some areas, and there may be restricted entry to privately owned land or reserves. Agricultural and other land use practices may make some areas unsuitable for sampling. Collaborative sampling with other projects, or obtaining samples from existing museum collections with sufficient metadata [11,51,63–65], can be cost-effective and efficient alternatives to field collection, specifically for the purpose of isoscape development.

Issues of scale and resolution are of central concern in landscape-level studies. Any interpolation method can be used to create isoscapes at any scale. Global or continental-scale isoscapes (e.g. [18,65]), that rely heavily on geospatial statistics and global covariate data are valuable as preliminary tools, including assessing whether the Sr isotope system is likely to help answer a given research question at any one locale. Many broad-scale isoscapes are, however, limited by the availability and geographically biased distribution of bioavailable ^87^Sr/^86^Sr data globally, as well as the relatively low resolution of global covariate data. As such, global models will usually require recalibration using local, potentially higher-resolution empirical and covariate information for application at regional and local scales. The required isoscape resolution is challenging to determine. For example, trying to provenance a butterfly which developed from a caterpillar that lived on a single plant is very different from tracking large mammals feeding over much bigger areas.

Ideally, one might wish for isoscapes based on covariate data and samples collected at a level of resolution relevant to the research questions about mobility or migration. Areas with few bioavailable ^87^Sr/^86^Sr data points or substantial geological complexity will require more intensive sampling. Heterogeneous felsic or metamorphic lithologies often tend to show greater ^87^Sr/^86^Sr variability, compared with more homogeneous carbonate rock types such as chalk and limestones [18]. Evans *et al.* [66] (as cited in [17]) found radiogenic igneous rock to have approximately 0.5% (0.0012–0.0036 per mil) 1 s.d. reproducibility, compared with approximately 0.05% (0.0004–0.0008 per mil) for carbonates.

When collecting samples, it is recommended to think beyond the current project, considering possible future use of the data by researchers interested in different questions. For each sample, it is important to record the GPS coordinates, elevation and geology or lithology, both from the published geological map of the area and actual field observations from the site. Due to limitations of scale and resolution, bedrock shown on a geological map may be draped by a thick layer of sediment or capped by a secondary deposit such as calcrete or ferricrete, and these observations are important to note. Apps in development to aid the collection of sample metadata will automatically fill out a number of these fields.

## 4. Are plants the best samples for isoscape creation?

The choice of material for empirical field measurements of ^87^Sr/^86^Sr depends on what is available in the area of interest and on the temporal and geographical scale of the research question. Most isoscapes are built using soil, plant, faunal or water samples, or a combination of these sample types. Terrestrial isoscapes differ from river isoscapes.

Some isoscapes are based on ^87^Sr/^86^Sr measurements of soil samples, but it can be difficult to estimate bioavailable ^87^Sr/^86^Sr from soils. Soils usually include resistant (less soluble) and more soluble components, so complete dissolution of bulk soils in strong acid yields total ^87^Sr/^86^Sr, which may be substantially different from the more soluble bioavailable signal taken up by plants and ultimately animals [34]. For the production of isoscapes, therefore, researchers typically leach soil samples to extract the more mobile (approximately equivalent to bioavailable) strontium fraction. The ^87^Sr/^86^Sr obtained is influenced by the leaching protocol (i.e. the reagent used, its concentration, duration of leaching, etc. [39,67,68]). These are examined in more detail in the sample preparation section. Some researchers report close correlations between ^87^Sr/^86^Sr of soil leachates and plants on a large scale [46,69], but several participants in the workshop have found soil leachates to yield such inconsistent results (e.g. [70]) that they now prefer other sample materials for estimation of bioavailable ^87^Sr/^86^Sr. In addition, ^87^Sr/^86^Sr in soils is highly susceptible to the effects of recent agricultural practices, such as the addition of lime [37,38,40,69], bone meal or basalt rock dust [71] used to supply minerals. Some organic fertilizers contain negligible quantities of calcium and strontium, so will not affect soil ^87^Sr/^86^Sr. Organic-rich fertilizers tend to increase soil δ^15^N, thus providing a marker of their presence [72–75]. Soil amendments will need to be taken into account if the intention is to provenance agricultural products [13] or apply ^87^Sr/^86^Sr in modern forensics [51].

Plants are generally considered a good material for estimating bioavailable ^87^Sr/^86^Sr [28,31,36,42,63], although they may be scarce in arid environments. As plants are primary producers at the base of the food web, patterning in plant ^87^Sr/^86^Sr will likely be a reliable proxy for ratios in consumers, although as organisms rooted in one spot, they may show more small-scale inter-individual variation than mobile animals. Any type of plant material (i.e. leaf, stem or bark, etc.) should be suitable and have similar ^87^Sr/^86^Sr [70]. Opinions differ on the importance of identifying sampled plants to species or at least genus level. There is no logical reason why ^87^Sr/^86^Sr should co-vary with taxonomy (as demonstrated by O’Regan *et al.* [76]), yet some researchers recommend recording plant species sampled [67]. In highly speciose biomes, identifying plants to species (and sometimes even genus) level can require the presence of flowers or fruit, which severely constrains sampling options. However, the depth and extent of root systems can vary substantially between species and between small and large specimens of the same species. It is therefore recommended to provide an estimate of plant type and rooting depth (shallow, medium, deep), as plants with different rooting depths may access different Sr sources in the soil [28,36,39,77–80], although this does not apply in all environments [47]. Attempts to characterize the bioavailable ^87^Sr/^86^Sr of the ecosystem as a whole should factor this into the collecting strategy and include both shallow- (e.g. grasses), medium- (e.g. shrubs) and deep-rooted plants (e.g. trees). Pooled samples from multiple plant species will generate an averaged signal [4,63,81] and reduce point bias [17]. A more targeted sampling strategy may be preferred for some research questions (e.g. if investigating organisms that live on specific plant species, like monarch butterflies, or that consume either deep- or shallow-rooted plants, such as browsing versus grazing herbivores [11,36]). Soil pH and cation exchange capacity can covary with ^87^Sr/^86^Sr [18,45]. O’Regan *et al.* [76] investigated whether mycorrhizal associations influence plant ^87^Sr/^86^Sr because mycorrhizae can change the pH of soils and thus influence the dissolution of minerals [82,83]. They found no significant differences between plants with and without associated mycorrhizae, although this may vary in different geological substrates, growing conditions and plant taxa sampled.

In some parts of the world, exotic dust can make a substantial contribution to the strontium budgets of plants [28,84]. Plant ^87^Sr/^86^Sr can also be impacted by anthropogenic alterations to the soil. If these are not relevant to the research question (i.e. the isoscape is not designed for a modern or forensic use-case), careful choice of sampling locality—avoiding areas currently or recently cultivated—can mitigate the problem. In general, the importance of fertilizer use for plant ^87^Sr/^86^Sr will need to be assessed on a case-by-case basis, including the possible influence on adjacent uncultivated areas [37,38,40].

Faunal samples (e.g. animal bones or teeth) have the advantage (compared with plants) of integrating ^87^Sr/^86^Sr over the animal’s feeding range, rather than providing spot values. Small terrestrial wild or commensal species such as rodents, with relatively small territories and short life-spans, have been used to estimate ‘local’ ^87^Sr/^86^Sr values, with both modern [1,64,85,86] and archaeological fauna [87–89]. However, one must ensure that such animals are local and not deposited a long distance away from their home range by an owl, for example [90]. Animals with large home ranges, correlated with larger body size [91], may incorporate Sr from multiple regions with different ^87^Sr/^86^Sr, making them unsuitable for constructing isoscapes, but see [92]. Some studies have shown overall consistency between species with small foraging ranges and plants (e.g. [29]), while others have not (e.g. [93]), highlighting the context-dependent nature of strontium propagation. In regions that have been intensively farmed for a long time, with a commensurate degree of soil modification, palaeo-studies may require isoscapes based on historical or archaeological materials [87].

A number of studies have analysed snail shells, reasoning that these contain significant amounts of strontium, are straightforward to collect, will yield reliably localized signals and are easy to process in the lab. It appears, however, that the ^87^Sr/^86^Sr of snails are strongly influenced by rainwater, probably because snails are heavily dependent on rainwater to generate slime [31]. If the ^87^Sr/^86^Sr of rainwater differs from the local geological substrate, snails may not be a reliable proxy for local bioavailable values. Other studies have found snails to track ^87^Sr/^86^Sr of shallow-rooted rather than deep-rooted plants [28], to be biased towards values for soil carbonates [39], and to yield reduced ranges of variation compared with local plants [36]. They may also prove difficult to find in certain areas (acidic soils, montane regions, etc.). All in all, snail shells appear sub-optimal for constructing isoscapes of bioavailable ^87^Sr/^86^Sr.

Water samples are easy to collect. Near a river’s source at a high elevation, water ^87^Sr/^86^Sr will resemble that of bedrock (at least, the fraction of bedrock susceptible to weathering). Downstream at lower elevations, the river may incorporate Sr from precipitation runoff through multiple geological formations with variable Sr contributions [24,34]. Although uncommon, freshwater ^87^Sr/^86^Sr can exhibit substantial temporal variation in environments with marked wet or dry seasons due to the alternating relative importance of surface runoff versus aquifer groundwater input [94]. Plants growing on floodplains near rivers have been found to have river-derived ^87^Sr/^86^Sr signals that differ from surrounding geological substrates [34,36]. Developing isoscapes of river systems requires the use of dendritic network models to adequately account for their complexity. These are discussed in detail elsewhere; see [95]. While the importance of Sr inputs from drinking water in consumer Sr budgets will vary with the relative concentration of Sr in the water versus that in the food, it is typically less impactful than food [96], suggesting that the collection of waters can be complementary but not necessarily a core requisite in bioavailability sampling studies.

## 5. How should samples be prepared and analysed?

The different sample types discussed above require different preparation for analysis. How samples are prepared will also depend on how Sr will be introduced into the mass spectrometer: typically, either in solution or via laser ablation with a multi-collector inductively coupled plasma mass spectrometer (MC-ICP-MS) or in solid form with a thermal ionization mass spectrometer (TIMS). Both TIMS and solution-based MC-ICP-MS offer high precision but involve a significant amount of sample preparation, typically acid digestion of ashed, powdered or bulk samples in a cleanroom laboratory, followed by Sr isolation using ion exchange chromatography (e.g. [97,98]). Although Sr is a trace metal, it is ubiquitous in the environment, including in unfiltered air. In the US, the concentration of strontium in urban air was on average 20 ng m^−3^ in the 1970s and has increased with growing air pollution [99,100]. The isolation of small amounts of Sr therefore requires a cleanroom laboratory to avoid atmospheric dust and avoidance of unnecessary exposure to metal surfaces, frequently a source of Sr contamination. Class 1000 or ISO 6 certified laboratories should indicate a sufficiently uncontaminated space. However, for a more thorough assessment of potential contaminants in the lab environment and in reagents, procedural blanks should be included in each batch of samples or at least measured at regular intervals. Sr concentrations in blanks below 1% of the values in samples being analysed indicate a clean work environment and process.

### 5.1. Sample measurement metadata

Metadata recorded along with analytical results should include the laboratory where the measurements were conducted and the protocols used during sample preparation. Laboratories should report the long-term, 2*σ* external reproducibility of their repeated analyses of a matrix-matched standard with Sr concentration similar to the samples (i.e. the 2*σ* standard deviation of the facility). Along with the number of analyses, this enables the calculation of the standard error if desired. Suitable standards include US Geological Survey BCR-2 [101] and BHVO-2 [102] for rocks and soil samples, NIST SRM 1400 and 1486 for bone and tooth samples [103], and NIST SRM 1515 for plant samples [104,105]. Alongside this, the standard error of the reference materials run during a particular project should also be reported. The reference value used in the analytical facility for NIST SRM 987 to which ^87^Sr/^86^Sr measurements are referenced should also be quoted (e.g. 0.710255 from Waight *et al.* [106]) to enable different datasets to be renormalized to the same value for direct comparability. Note that the value of 0.71034 ± 0.00036 originally reported for SRM 987 [107] is outdated, and a large, improved set of values from many facilities is available [108]. Values for laboratory procedural blanks, the magnitude of blank elemental Sr, results for known unknowns analysed alongside project samples, and the numbers of repeat analyses should also be reported. Finally, it is helpful to include strontium concentrations of samples, if available.

### 5.2. Soil samples

For isoscapes of bioavailable ^87^Sr/^86^Sr, soils should be leached to isolate the bioavailable and thus biologically meaningful Sr fraction, which is water soluble and exchangeable. As mentioned above, different leaching protocols yield different ^87^Sr/^86^Sr, and there is little consensus on which are best [68]. Published studies variously report leaching with water alone [39], nitric, acetic [46,69,109] or hydrochloric acid [110] of varying concentrations (0.1–1 M) or 1 M ammonium nitrate solution (NH_4_NO_3_) [111] following the protocol DIN International Organization for Standardization (ISO) 19730 [112]. Some studies have compared multiple methods [13,67,68,113,114]. The leachates are then separated using centrifugation [115] or by filtering through a syringe or pipette equipped with a membrane [51], dried down, and Sr is isolated using ion exchange column chromatography. For the sake of data reproducibility and metadata analysis, authors should publish details of the protocol used for leaching.

### 5.3. Plant samples

Once collected, plant samples should be stored in unbleached, lightly closed paper bags and allowed to dry out completely to avoid the formation of mould. If long-distance (international) transport is necessary before analysis, it may be advisable to first char them (if they will ultimately be ashed), to avoid the possible need for plant importation permits. In the field, plants can be wrapped in aluminium foil and left near a fire, allowing them to turn into charcoal [116]. Alternatively, plants can be frozen at −80°C for 72 h [28] or gamma-irradiated [115] to destroy seeds and pests.

Some studies have rinsed plants with deionized or ultrapure water before analysis to remove surface dust [11,29,36,117], while others have not, presumably (although not necessarily explicitly) reasoning that atmospheric Sr deposition contributes to bioavailable ^87^Sr/^86^Sr ingested by consumers feeding on plants [4,63]. In a study in the UK, Warham [70] found that unwashed leaves and inner woody material from the same trees, Warham yielded the same ^87^Sr/^86^Sr.

Field-collected plant samples are usually sub-sampled for processing in the laboratory. To ensure the recovery of sufficient amounts of Sr, we recommend preparing at least 1.5 g of dry plant matter or approximately 20 mg of ash. In the laboratory, plants are either ashed or treated with strong oxidizing agents (e.g. hot nitric acid) to remove organic compounds that can interfere with the separation of Sr on resins used in column chromatography. Increasingly, many laboratories use microwave digestion to achieve this more efficiently (e.g. [62]). A typical ashing protocol is as follows: the dry material is cut or crumbled into small pieces (combining different specimens if desired) to partially fill pre-cleaned crucibles made of porcelain, ceramic or pure silica or quartz, leaving space for air to circulate. The crucibles are then placed in a muffle furnace with temperatures between 500°C and 800°C for about 10 h, depending on the laboratory. The resultant ash is homogenized in the crucible, transferred to a clean storage vial and is then ready for acid digestion and separation of Sr in a clean lab setting. If plants are to be analysed for other isotope systems such as carbon, nitrogen or sulphur, sub-samples must be removed before ashing. Another method is to dry the plants overnight in an oven at approximately 40°C, then homogenize into a fine powder before acid digestion, possibly using a microwave digestor [31].

### 5.4. Bone and enamel samples

Like plants, organic-rich bones are either ashed or treated with hot nitric acid. For calcium-rich materials such as bone, 10–30 mg will contain sufficient Sr for analysis, with similar preparation and Sr separation methods to those employed for plants as described above. Alternatively, hard, mostly inorganic sample materials, such as well-preserved tooth enamel, are suitable for analysis by laser ablation MC-ICP-MS.

Laser ablation needs minimal sample preparation and has high spatial resolution but relatively low precision. Corrections for isobaric interferences may be required (e.g. [118–120]). It is quicker and less destructive than solution ICP-MS or TIMS since it does not require separation of Sr [121]. The method is minimally destructive for small samples (e.g. individual human teeth) that can be placed in the laser chamber; larger ones (e.g. large animal teeth) may have to be sub-sampled. Laser ablation requires samples to have relatively high concentrations of Sr and low Rb (since Rb cannot be removed before analysis and the ^87^Rb isobaric interference has to be corrected, although future technological developments may help to mitigate this issue). The 2*σ* external reproducibility (two s.d. values of the mean value of multiple repeated analyses of the same sample) of ^87^Sr/^86^Sr measurements by laser ablation is typically 0.0002–0.0003 [122], larger than for solution analyses (where the uncertainty is typically in the fifth decimal place). Uncertainty in the fourth decimal place may be perfectly adequate given the numerous sources of variation in plants and animals. Laser ablation allows spatially highly resolved analyses of targeted areas of a sample, enabling the investigation of (for example) changes throughout tooth formation, or focus on areas with the best preservation. This is more difficult with solution methods, which require targeted enamel removal before digestion. If laser ablation is to be used, care should be taken that any cleaning of the sample will not compromise the isotopic measurements. Experience in the MC-ICP-MS Facility in Geological Sciences at the University of Cape Town indicates that wiping the surface of the tooth with acetone before analysis is problematic, since acetone leaves behind an organic residue. Ethanol or methanol does not, so is preferred for this purpose. This lab has also found that juvenile teeth are often not suitable for laser ablation, likely due to less mineralized enamel. While undertaking measurements on flat surfaces (sectioned or sliced samples, or even polished thin sections) is generally preferred, analyses of slightly curved surfaces can produce reliable data combined with mass correction [123].

Ancient (archaeological, palaeontological, some historical) samples may have undergone diagenetic changes that compromise our ability to measure biogenic ^87^Sr/^86^Sr. Understanding diagenesis comprises a substantial field of study in its own right [124,125], which is beyond the scope of this article, although there is one near-consensus point: bone is far more vulnerable to diagenesis than tooth enamel [126]. Unless calcined [127], or modern without prolonged contact with the soil [128], bone is best avoided as a sample material for ^87^Sr/^86^Sr. With regards to possible pre-treatment protocols, we note that methods designed to remove one diagenetic component (e.g. carbon or oxygen) from calcified tissues do not necessarily remove others (e.g. strontium). Oxygen occurs in calcified tissues in various chemical forms (carbonate, phosphate, in the hydration layer), some structural and others only loosely bound and hence readily exchangeable. Strontium, on the other hand, generally substitutes for calcium in the crystal lattice of bioapatite. Crowley *et al.* [129] found measurable differences in ^87^Sr/^86^Sr between samples of powdered tooth enamel prepared using different chemical pre-treatments. However, they cautioned that possible contamination introduced in the laboratory could not be ruled out [129]. Removing the outer layers of tooth enamel before analysis can help eliminate diagenetically altered material. Protocols for measurement of ^87^Sr/^86^Sr by laser ablation typically do this using a rapid pre-ablation cleaning laser sweep along the intended path of analysis with a slightly larger spot size to remove the outer few microns of enamel (typically 2−5 μm) [123].

## 6. What are the complications with assigning provenance?

Once an isoscape has been created, there are multiple ways to use it for geographically assigning samples of unknown origin. The simplest is visual comparison to match the ^87^Sr/^86^Sr of the unknown to the different zones on the isoscape. There are also GIS-based tools [130] and probabilistic approaches such as the *assignR* package in R [131], which also allows for the incorporation of additional constraints from other isotopic or non-isotopic data. The term ‘assignment’ is somewhat misleading, as the identified, assigned location simply represents the area of most probable origin. Bayesian probabilistic approaches like that implemented in *assignR* do not generate areas of zero probability (i.e. there is always some possibility of any particular locality being the origin).

The isotopic composition of consumer tissues reflects combined Sr inputs from various sources over a period of time. The more mobile the individual, and the more heterogeneous the landscape, the more sources will be incorporated. In complex cases, the consumer may match none of the contributing sources, or may yield a value compatible with more than one combination of sources, termed equifinality.

In regions of relatively homogeneous ^87^Sr/^86^Sr, such as many carbonate-based geological substrates, variations between consumers in the fourth decimal place can be meaningful [66]. In areas of more heterogeneous lithology and mineralogy, variations in the fourth place may merely be noise. Assignment can be refined with the use of contextual knowledge such as the topography of the area, rates of erosion and reworking of a landscape, known migration patterns of the species studied and/or combining with other isotopic tracers. However, one should be conscious of the influence of this prior information on the assignment. Incorrect assumptions may substantially bias or compromise the interpretations drawn or result in circular or confirmatory logic.

## 7. What are the influences of biology and metabolism on consumer tissue Sr?

Sr isotope provenancing of organic materials lies at the intersection between biology and geology. The geological aspects are relatively well understood, but much less attention has been paid to the biology. It has long been known that strontium is incorporated into vertebrate calcified tissues because it substitutes for calcium, replacing it in bioapatite [132,133]. Absorption of alkaline earth elements in the mammalian digestive tract favours calcium and discriminates against Sr [132,134], so the extent of Sr incorporation is proportional to [Sr]/[Ca]. Foods with high [Sr]/[Ca] (e.g. plants and dairy foods) contribute more Sr to consumer tissues than those with low [Sr]/[Ca] (mainly non-dairy animal foods) [42,135,136]. Vitamin D-rich foods promote dietary Ca absorption; this may apply also to Sr [133]. The biopurification process results in reduced variance in [Sr]/[Ca] and ^87^Sr/^86^Sr as food sources are averaged up the food chain [42]. In Britain, the interquartile range of variation seen in ^87^Sr/^86^Sr in archaeological human tooth enamel is approximately half that of plants, although the means and medians are similar [137]. Relatively little attention has been paid to [Sr]/[Ca] in isoscapes (although see [15]), but if these vary systematically across the landscape, we might expect to see differential contributions of foods from different regions (i.e. unequal visibility of various regions) in consumer ^87^Sr/^86^Sr. Lewis *et al.* [19] found that [Sr]/[Ca] in pig tissues increased with a greater proportion of marine food (fishmeal, which likely included small bones). We note also that the concentration of Sr in seawater is typically 8 ppm [26], compared with <1 ppm in rainwater [32]. The proportion of marine-derived Sr incorporated into the diet in the form of seafood or salt can influence ^87^Sr/^86^Sr in tissues and potentially skew provenance studies if unaccounted for [133,138]. Ingested dust influences consumer tissue ^87^Sr/^86^Sr [139], although gritstone, sandstone and granite grindstones used in grain processing appear to have little effect [140].

Details of the rates and processes of Sr uptake in different species, and mobilization and recycling of Sr already in the skeleton (especially in relation to nutritional status and pregnancy and lactation) are not well understood. Only a few, relatively small studies have analysed animals fed controlled diets. The patterns reported thus far are inconsistent: inter-individual variation in ^87^Sr/^86^Sr among animals fed isotopically monotonous diets ranged from the third decimal place (0.001) for guinea pigs [139] to the fourth decimal place (0.00062) for cattle [141] and the fifth place (0.00002–0.00008) for pigs [19] (but see [142]). Weber *et al.* [139] found larger inter-individual differences in guinea pigs compared with rats fed on the same diets. Inter-species differences may derive from different digestive efficiencies, perhaps linked to different pH in the digestive tract. It is critically important to know how much of the ^87^Sr/^86^Sr variation we see in our unknowns might result from within-animal biological factors alone. We can then estimate the threshold above which differences in ^87^Sr/^86^Sr are attributable to different diets and/or places of residence. Further multigenerational controlled feeding studies with larger numbers of animals are urgently needed to improve our understanding of this issue.

^87^Sr/^86^Sr in body tissues reflects food and drink consumed at the time of tissue formation, buffered by ^87^Sr/^86^Sr already circulating within the body. Even rapid movement to very different lithologies, resulting in a sudden change in dietary ^87^Sr/^86^Sr, will lead to a relatively gradual change in tissue ^87^Sr/^86^Sr due to the dilution of newly ingested Sr by the available body pool [143,144]. This effect is well-known for stable light isotopes (e.g. [145]), and additional studies on ^87^Sr/^86^Sr turnover may lead to the development of models to correct for this process in specific taxa (e.g. [144]). In mammals, bone mineral from the mother’s skeleton is resorbed and incorporated into the metabolic pool during lactation, so ^87^Sr/^86^Sr in milk, and calcified tissues forming in suckling infants, reflect a mixture of the mother’s current food and drink and that dating from earlier in her life. In humans, mothers typically lose 2–8% bone density during lactation [146], and some studies report that maternal skeletal turnover may be as high as 10–30% during pregnancy and lactation [147,148]. The implication is that ^87^Sr/^86^Sr of calcified tissues formed in early life will be influenced by diet and place of residence over more than one generation. Although the contribution of maternal Sr to infants is well known, the implications for provenance have not been adequately addressed.

## 8. Do specific applications require custom isoscapes?

The degree to which specific applications (such as provenancing animal species with different behaviours or various types of materials like glass, ceramics or eggshells) require different isoscapes probably needs to be decided on a case-by-case basis. For example, isoscapes of bioavailable ^87^Sr/^86^Sr may be suitable for assessing the origins of materials such as glass in which Sr comes mostly from wood ash [149,150], and for ceramics if geology is a major driver of ^87^Sr/^86^Sr variation in a particular study area [151]. One view is that researchers should not re-use existing isoscapes for different purposes, but rather create new ones tailored to specific research questions using the same freely available data. It is valuable to create a flexible, adjustable isoscape in which different types of samples can be included or excluded to tailor the output to a particular research question. For example, if ^87^Sr/^86^Sr varies significantly and consistently with rooting depth, it might be desirable to develop separate isoscapes for grazing and browsing animals, based on shallow- and deep-rooted plants, respectively. The *calRaster* function in the *assignR* package is one potentially useful tool in this regard: it can convert isoscapes developed with one type of material (e.g. plants) into versions suitable for other applications by re-calibrating with a small number of known origin samples of the second type (e.g. butterfly wings, ratite eggshell or dental enamel). The function uses linear modelling and assumes that a systematic, linear relationship exists between the existing isoscape and the target sample material (although this may not always be the case for ^87^Sr/^86^Sr). If the relationship is chaotic or not a linear function of the existing isoscape values (visualized by the generated plots and statistics), *calRaster* will not improve the accuracy of the isoscape. However, it will still propagate the uncertainty inherent in the comparison between the sample substrate and the isoscape, giving a more realistic estimate of uncertainty for use in subsequent assignment calculations. It is important to recognize when this is the case and adjust your analysis accordingly. For example, a very weak relationship between the isoscape and the target sample values could mean that the selected isoscape is a poor representation of the study system. One approach would be to evaluate other isoscapes (e.g. generated using different data or methods) to figure out whether they perform better. Alternatively, a weak relationship may also demonstrate more fundamental limitations of the method. For example, in the case of contemporary North Americans studied by Verostick *et al.* [152], the low slope and noisy relationship shown in their fig. 3B probably reflect (in large part) the isotopic homogenization of Sr sources in contemporary humans through globalization of diet and individual travel. In these cases, it is important to recognize the aspatial variation and factor it into assignment analyses.

In a comparison of ^87^Sr/^86^Sr isoscapes of Western Europe based on soils, plants, and surface waters, Bataille *et al.* [45] reported offsets in the third decimal place. This is not surprising given that there are difficulties with assessing bioavailable ^87^Sr/^86^Sr in soils, as discussed above, and the patterning of ^87^Sr/^86^Sr in aquatic systems differs from that of terrestrial systems. In the southern Andes, Barberena *et al.* [93] reported a higher correlation between measured and predicted ^87^Sr/^86^Sr for an isoscape based on rodents compared with one based on both rodents and plants collected from the same landscape. Variation in rodent ratios was driven by a smaller number of variables, mostly related to geology, compared with variation in plants, for which several bioclimatic variables played a role. As the authors point out, in this case, the faunal isoscape is likely to be better suited to provenancing archaeological fauna and humans than the plant isoscape. However, the sample sizes used in this study were relatively small, given the large study area.

## 9. What are the remaining uncertainties and limitations of strontium isoscapes?

Regardless of the details of isoscape creation, robust and honest quantification of the uncertainties in the isoscape predictions is critical when evaluating the origin of unknown samples using probabilistic methods [153,154]. In these approaches, the predicted isoscape value (mean prediction and uncertainty) at any given location is used to estimate the distribution of ^87^Sr/^86^Sr at each location of the isoscape. By comparing the ^87^Sr/^86^Sr of a sample of unknown origin to the estimated ^87^Sr/^86^Sr distribution at each site, one can assess the likelihood that this sample might have come from any given location on the isoscape. Overly optimistic uncertainty estimates will give the impression that the sample origin is more tightly constrained than it really is, while pessimistic estimates will underrepresent the strength of the isotopic assignment. A common error is to confuse uncertainty estimates of the mean isoscape predictions with the variance expected among individuals originating from a common location. As we are almost always seeking to constrain the origin of individuals, isoscape uncertainty estimates should ideally incorporate the variability between individuals within a local population. This is currently not standard practice for ^87^Sr/^86^Sr, although it is for ^18^O/^16^O and ^2^H/^1^H isoscapes in which researchers frequently analyse multiple known-origin specimens from the same location (e.g. [155,156]). Different methods exist for generating robust uncertainty estimates, ranging from analytical propagation of component uncertainties to estimating ‘bulk’ uncertainty by comparing system-specific, known-origin data with isoscape predictions (as is done in the *calRaster* function discussed above, for example [153]). Each method has benefits and limitations, which are beyond the scope of this review, but isoscape developers should report how uncertainty was estimated, and users should assess the adequacy of the estimates for their application.

To illustrate the uncertainties associated with the predictive modelling approach, isoscapes should be accompanied by standard error maps. Higher predictive standard error in some regions may be the result of low sample size, skewed sample distribution, spatially heterogeneous predictor variables or a combination of these factors. These prediction error maps are particularly important when using isoscapes to assign provenance of a given specimen (e.g. in *assignR*), allowing a degree of assignment uncertainty integration. Areas with high prediction errors can also help identify regions with poorer sample coverage for targeted future sampling efforts. However, standard predictive error maps may not be the only tool to identify areas that require local re-sampling or cautious use; in their strontium isoscape of sub-Saharan Africa, Wang *et al.* [65] employed multivariate Mahalanobis distance as an indicator of environmental dissimilarity, to identify regions with unusual environmental (predictor variable) conditions not well represented by the training dataset [157].

A number of uncertainties and limitations associated with Sr isoscapes remain. They include uncertainties associated with the covariates, such as the accuracy of geological maps and model calibration for the past (e.g. changes in the position of shorelines, shifts in vegetation cover, changes in surface geology like alluvium deposition and other significant natural or human modification of the landscape). There are also uncertainties in the development of isoscapes, including how to manage regions without training data. Should one extrapolate into unsampled areas, or should they be left blank to be filled in by later studies? Extrapolation will produce predictions with increased uncertainty and runs the risk of an end-user being overly confident in the results. The decisions surrounding the risks of extrapolation can, of course, be case-specific, but leaving areas blank is advisable given that random forest cannot reliably extrapolate ^87^Sr/^86^Sr predictions into unsampled areas [158]. This is particularly true for continental-scale isoscapes with large spatial gaps between datapoints and highly heterogeneous environmental conditions, but possibly less relevant for comparatively focused isoscapes in homogeneous landscapes. Some areas may be less likely candidates for activities such as ancient human land use, based on the historical or archaeological record or even the topography. A large sedimentary basin or area of loess may have significantly less variability in ^87^Sr/^86^Sr across a large area than a mountain range, but may represent a more likely area of use by agriculturalists.

### 9.1. Sources of error

There are many possible sources of error during the process of sample collection and analysis. Errors can occur at the point of initial sample collection (e.g. mistakes in animal species identification, plants growing in unusual microhabitats) or during subsequent preparation (e.g. inadequate homogenization). Contamination of the sample, such as with solvents used as cleaning agents, can occur before or after the sample arrives in the lab and this can be challenging to detect. Instrument errors can result from (for example) low Sr concentrations, or interferences. It is important to analyse matrix-matched known unknowns (i.e. test samples of materials similar to the samples) and choose appropriate international and in-house standards to monitor, assess and minimize such errors.

## 10. Where to from here?

### 10.1. Adding other isotopes

Including other isotope systems alongside ^87^Sr/^86^Sr is useful for provenance studies and should increase the accuracy of assignments [159] and may expand the current boundaries of the field by making visible previously unforeseen connections. Oxygen (^18^O/^16^O, expressed as *δ*^18^O) and hydrogen (^2^H/^1^H, expressed as *δ*D or *δ*^2^H) show gradients with precipitation [153,160,161]. Patterning in sulphur (^34^S/^32^S, expressed as *δ*^34^S) is more complex, but applications include proximity to the coast and identifying wetland inhabitants [162–164]. Lead isotopes can help to refine likely origins when ^87^Sr/^86^Sr is not definitive e.g. [165]). Pb derives from both natural geological sources and anthropogenic ones, such as lead pipes and leaded petrol; the extensive use of Pb-rich materials in the Roman Empire is well-known [166,167]. It may be easy to identify contamination by anthropogenic sources. Millard *et al.* [168] found that if samples have Pb concentrations below 0.87 ppm, there is little risk of anthropogenic contamination, so isotope analysis is likely to provide meaningful clues to provenance. Both Pb and Sr can be isolated from the same sample, although the very low concentration of Pb in human teeth means that reliable analyses require relatively large sample sizes. The precise relationship between geological and bioavailable plant Pb has not been closely investigated, but because animals take up Pb mostly via accidental ingestion of soil or dust [169,170], rather than from food, isotope measurements for Pb-containing minerals are more usable as a tracing tool, and there is less need for mapping of bioavailable Pb isotope ratios than there is for Sr. Neodymium isotopes (^143^Nd/^144^Nd) show an inverse relationship with Sr isotopes. The mammalian gut discriminates strongly against the absorption of rare earth elements, so there are very low concentrations of Nd in mammal teeth [171]. Neodymium isotopes may therefore be less helpful for provenancing humans or other mammals, but potentially useful for materials such as ratite eggshells.

In addition to the use of other isotope systems to bolster interpretations based on ^87^Sr/^86^Sr, strontium isotopes are also important in reinforcing or challenging inferences drawn from other isotope datasets in archaeology and palaeoecology. ^87^Sr/^86^Sr can be used to trace or exclude non-local provenance and past mobility. For example, studies reconstructing regional palaeotemperatures based on *δ*^18^O of ungulate teeth have also analysed ^87^Sr/^86^Sr on intra-tooth oxygen isotope peaks and troughs to exclude the possibility that the animal was migratory; we can thus be confident that the *δ*^18^O values reflect ‘local’ seasonal climatic variation (e.g. [172]). Similarly, *δ*^66^Zn analysis, a relatively new method of reconstructing trophic level in ancient food webs from tooth enamel, may increasingly be paired with ^87^Sr/^86^Sr to ensure that variability in zinc isotopes reflects trophic level rather than variations in bedrock composition [173,174].

### 10.2. Isoscapes for forensics

The application of isotopes for provenance in forensics has garnered substantial interest, including the use of multiple isotope systems to assist in reconstructing residence and travel history for the identification or repatriation of human remains [9,175], and sourcing of illegally traded wild plants [176] and animals [43,177,178]. The value of ^87^Sr/^86^Sr is probably limited in urban human populations that buy and consume internationally sourced foods from supermarkets (although see [179]); it is likely to be more useful in the case of rural subsistence farmers. Strontium isotopes also play a role in ‘food forensics’ [180] to demonstrate the geographical origins of food products, sometimes required for food authentication. For example, ^87^Sr/^86^Sr has been shown to be useful in differentiating the origins of European wines (e.g. [181]), allowing the detection of counterfeit wines [182]. Along with other isotope systems, strontium isotopes are, however, used mainly as a forensic investigative tool (rather than as evidence in court) because of the limitations of their inherent uncertainty. Isotope approaches can only identify areas of probable origin or exclude other unlikely regions. Definitive proof of provenance requires additional types of forensic evidence. Nevertheless, forensic studies incorporating isotopes are a growing focus. It is worth noting that for forensic purposes, agricultural and industrial alteration of landscape ^87^Sr/^86^Sr will need to be explicitly incorporated into isoscape design, instead of being excluded as is usually the case in archaeological or other palaeo-studies.

### 10.3. Data repositories

As the quantity of isotopic data in the literature grows, long-term open-access storage, making data available and accessible for multiple different uses, is a goal for the field as a whole. There are multiple repositories for isotope data (e.g. [183,184]); we highlight two. IsoBank is a multi-disciplinary repository founded in 2017 with funding from the US National Science Foundation [185]. It is USA- and Canada-focused but is expanding to other regions [186]. There is a strong focus on metadata requirements to promote usage in multiple, diverse applications, and there are plans to create database interfaces that will allow data to be accessed and used directly in scientific software packages that enable isoscape development and analysis. The second, IsoArcH, is an archaeology-focused, community-driven platform that stores isotopic data for academic purposes with standardized metadata definitions and language, shared using the FAIR and CARE principles [187,188]. It was originally focused on Europe and, to a lesser extent, Asia, but has expanded to incorporate over 65 000 measurements from all over the world. An excellent map function shows the geographical distribution of data. It is an open-access repository where one can create private projects with a DOI and multiple collaborators that can instantly go public upon manuscript acceptance. It requires donations to keep it running, which can be built into grant budgets. Initiatives are underway to compile and harmonize existing published bioavailable ^87^Sr/^86^Sr data for incorporation into these repositories, to provide researchers across many disciplines with a foundation for their individual research projects. Researchers are encouraged to engage with these platforms, making use of currently available data and uploading newly acquired data directly. These repositories also include valuable lists of references to published studies. Since these data repositories are open-access, there is a chance that data may be misused to produce research of questionable scientific value or that contributors to the repository may not be given proper credit. Therefore, robust peer review remains critical to achieve quality publications and ensure correct citation of the papers where the data were originally published, not just the repository itself.

### 10.4. Ethics and accessibility

The use of isotope analyses for investigating diet, provenance, mobility and migration can raise ethical concerns. These require consideration to ensure we work mindfully in the academic and non-academic spheres in which our research is relevant [189]. Additionally, there are ethical challenges with publishing forensic data, such as the locations of endangered plant or animal species, at risk of being exploited.

Isoscape maps should be developed using colour palettes that, where possible, are legible for people with red–green colour vision deficiency, and we should use different shapes or sizes for data points in graphics, rather than relying on colour alone to distinguish them. Various tools and palettes have been developed to support the visualization of different types of data, and many of these can be accessed directly in scientific software environments like R and Python. The *colorbrewer* tool and libraries (colorbrewer2.org) have several useful colour palettes, and COBLIS (color-blindness.com/coblis-color-blindness-simulator) is a useful website that simulates how a colour-blind person would see an uploaded figure.

It is a concern that many researchers around the world have little or unequal access to the expertise and facilities required to carry out ^87^Sr/^86^Sr and other isotopic analyses. Many regions have significant economic challenges that hinder exposure to and training in laboratory-based sciences. Nevertheless, there are opportunities to obtain funding for training and collaboration with established laboratories.

## 11. Conclusions

This article highlights the enormous potential of ^87^Sr/^86^Sr isoscapes to help address questions about the origin and movement of goods, people and animals in many different fields, including archaeology, ecology, forensics, wildlife conservation, food science and others. Major concerns for practitioners include the choice of approach to creating an isoscape. In many cases, domain mapping performs well, yielding user-friendly isoscapes. In complex situations, however, especially where there are multiple important strontium inputs, machine learning approaches (especially random forest) are probably best. These require significant developer skills and access to adequate computing capacity. The choice of samples to be collected will depend on what is available, but most researchers favour plants because they form the base of the food web and are widely available (except in extreme environments). For landscape-level isoscape construction, the most widely applicable approach to bioavailable ^87^Sr/^86^Sr determination is by sampling plants within defined geological contexts. Faunal remains (modern and/or ancient) can, where present, supplement and complement plants. We survey strategies and processes for sample collection, preparation and analysis. An important contribution of this paper is that it highlights the relatively understudied influence of biology and strontium metabolism on ^87^Sr/^86^Sr in consumer tissues, particularly with regards to dietary choices and gut absorption, and also maternal recycling of skeletal Sr during pregnancy and lactation and how this may influence the chemistry of the growing tissues of offspring. We discuss the limits of applicability of isoscapes, in terms of materials and uncertainties, and the estimation of those uncertainties and the role this should have in assignment and data interpretation. Researchers need to make their own choices about the issues explored here, depending on their field of study and research questions, but we hope that the insights offered here will guide practitioners towards better outcomes.

## Data Availability

Supplementary material is available online [190].
